# Modulation of the Dynamics of a Two-Dimensional Interweaving Metal–Organic Framework through Induced Hydrogen Bonding

**DOI:** 10.1021/acs.inorgchem.3c04522

**Published:** 2024-03-14

**Authors:** Pilar Fernández-Seriñán, Kornel Roztocki, Vahid Safarifard, Vincent Guillerm, Sabina Rodríguez-Hermida, Judith Juanhuix, Inhar Imaz, Ali Morsali, Daniel Maspoch

**Affiliations:** † Catalan Institute of Nanoscience and Nanotechnology (ICN2), CSIC and The Barcelona Institute of Science and Technology, Campus UAB, Bellaterra, Barcelona 08193, Spain; ‡ Chemistry Department of Autonomous, University of Barcelona (UAB), Campus UAB, Bellaterra, Barcelona 08193, Spain; § Faculty of Chemistry, Adam Mickiewicz University, Uniwersytetu Poznańskiego 8, Poznań 61-614, Poland; ∥ Department of Chemistry, Iran University of Science and Technology, Tehran 16846-13114, Iran; ⊥ ALBA Synchrotron, Cerdanyola del Vallès, Barcelona 08290, Spain; # Department of Chemistry, Faculty of Sciences, Tarbiat Modares University, Tehran P.O. Box 14115-175, Iran; ∇ ICREA, Pg. Lluís Companys 23, Barcelona 08010, Spain

## Abstract

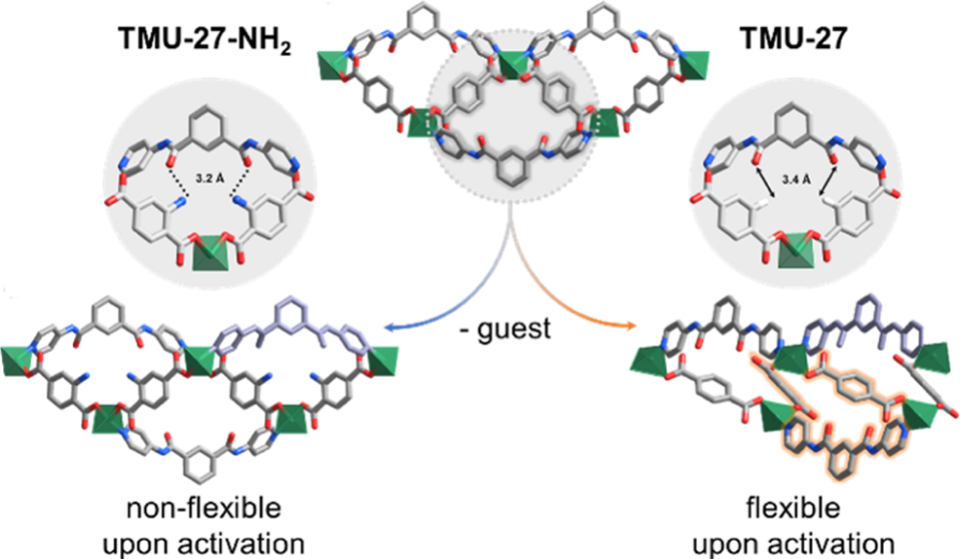

Inducing, understanding, and controlling the flexibility in metal–organic frameworks (MOFs) are of utmost interest due to the potential applications of dynamic materials in gas-related technologies. Herein, we report the synthesis of two isostructural two-dimensional (2D) interweaving zinc(II) MOFs, TMU-27 [Zn(bpipa)(bdc)] and TMU-27-NH_2_ [Zn(bpipa)(NH_2_-bdc)], based on *N*,*N*′-bis-4-pyridyl-isophthalamide (bpipa) and 1,4-benzenedicarboxylate (bdc) or 2-amino-1,4-benzenedicarboxylate (NH_2_-bdc), respectively. These frameworks differ only by the substitution at the meta-position of their respective bdc groups: an H atom in TMU-27 vs an NH_2_ group in TMU-27-NH_2_. This difference strongly influences their respective responses to external stimuli, since we observed that the structure of TMU-27 changed due to desolvation and adsorption, whereas TMU-27-NH_2_ remained rigid. Using single-crystal X-ray diffraction and CO_2_-sorption measurements, we discovered that upon CO_2_ sorption, TMU-27 undergoes a transition from a closed-pore phase to an open-pore phase. In contrast, we attributed the rigidification in TMU-27-NH_2_ to intermolecular hydrogen bonding between interweaving layers, namely, between the H atoms from the bdc-amino groups and the O atoms from the bpipa-amide groups within these layers. Additionally, by using scanning electron microscopy to monitor the CO_2_ adsorption and desorption in TMU-27, we were able to establish a correlation between the crystal size of this MOF and its transformation pressure.

## Abstract 2

Flexibility in metal−organic frameworks (MOFs) may be key for further advances in their industrial applications. Therefore, exploring what might trigger it is crucial, since even a minimal change within their structure, such as the substitution of an H for an NH_2_ group in the position of one of its constituent ligands, can influence the material’s behavior toward external stimuli as a whole.

## Introduction

Metal–organic frameworks (MOFs) are porous materials made by combining metal ions or clusters with organic linkers and can be designed for practical applications.^1^ There are countless available building blocks for MOFs, the nearly infinite combinations of which can yield novel materials with unprecedented structural and functional properties. The systematic study of structure–function relationships in MOFs has afforded presynthesis design strategies such as reticular synthesis^1^ or crystal engineering.^2^

Among the many ways to classify MOFs, one is by their structural flexibility, with relatively rigid MOFs representing a large group and flexible or dynamic-porous MOFs^3^ comprising a much smaller group.^4,5^ Here, flexibility refers to the dynamic responses of MOF structures to stimuli such as changes in temperature,^6−8^ pressure,^9,10^ light,^11^ and electrical fields^12^ or, most commonly, during host–guest interactions.^13^ Interestingly, MOFs respond to stimuli through diverse mechanical mechanisms such as breathing,^14^ swelling,^15^ linker rotation, or subnetwork displacement,^3,16^ all of which involve deformation of their pores and appear, on isotherms, as peculiar singularities.^17^ These mechanisms are dictated by three main factors: (i) the nature of the inorganic molecular building blocks;^13,18^ (ii) the rotational freedom of the organic linker;^19^ and (iii) the topology of the framework.^13^ Moreover, features stemming from the supramolecular nature of a MOF structure, such as intermolecular interactions within the structure itself or between the structure and the guest, must also be considered.^13,20,21^

Metal–organic frameworks typically boast excellent surface areas, tunable pore sizes, and physicochemical robustness, making them of great interest for practical applications.^22−28^ Importantly, adding or increasing the flexibility in MOFs can enhance their performance in various fields. For example, in catalysis,^29^ it allows for the formation of stimuli switching catalysts; in molecular sensing, it enables specific host–guest interactions;^30^ and in drug delivery, it improves control over conditional release.^31^ Additionally, flexibility can enable separation of water isotopologues, namely, via controlled diffusion through the dynamic aperture of pores,^32^ enhanced proton conductivity,^33^ and improved gas storage^17,34,35^ or separation^36^ through expanded storage capacities and potential selectivity.

While researchers have identified flexible MOFs and studied their mechanisms of transformation, relatively little progress has been made on inducing and controlling flexibility in MOFs or similar porous materials. A few recent examples have revealed that single-atom substitutions in organic linkers can alter phase transition mechanisms (e.g., from a stepwise transformation into a continuous one)^37,38^ or lead to changes in the isotherm type.^37−39^ Furthermore, there are reports that crystal size may influence the adsorption properties of flexible MOFs,^35,40^ which in turn might relate to shape-memory effects,^41^ negative gas adsorption,^42^ or even rigidification.^40^ However, before a priori design of flexible MOFs can be functional, a better fundamental understanding of the features that trigger and control their dynamic behavior is needed.^43,44^

Here, we report the design and synthesis of a novel flexible two-dimensional (2D) interweaving MOF with formula [Zn(bpipa)(bdc)]·2DMF (hereafter called TMU-27; bpipa = *N*,*N*′-bis-4-pyridyl-isophthalamide and bdc^2–^ = terephthalate) as well as of its more rigid counterpart, [Zn(bpipa)(NH_2_-bdc)]·2DMF (hereafter called TMU-27-NH_2_; NH_2_-bdc^2–^ = amino terephthalate). Note that these MOFs differ only by the substitution at the meta-position of their respective bdc groups: an H atom in TMU-27 vs an NH_2_ group in TMU-27-NH_2_. While the introduction of aromatic amines into MOFs usually has only a negligible to moderate impact on its sorption properties,^45,46^ TMU-27-NH_2_ is a rare example in which the impact is instead quite dramatic, as we report here. Indeed, by comparing the two isoreticular MOFs, in both the presence and absence of guest molecules in their pores, through a detailed structural analysis by single-crystal and powder X-ray diffraction (SC- and PXRD) and CO_2_-sorption measurements (taken at 203 K), we observed that TMU-27 is flexible upon CO_2_ sorption/desorption and that this flexibility is blocked in TMU-27-NH_2_ by the introduction of a single functional amino group in the organic linker. Additionally, through monitoring of repetitive CO_2_ adsorption and desorption in TMU-27, using scanning electron microscopy (SEM), we were able to establish a correlation between its transformation pressure and its crystal size.

## Experimental Section

### Synthesis of bpipa

Isophthaloyl chloride (1.015 g, 5 mmol) was slowly added to a solution of 4-aminopyridine (1.882 g, 20 mmol) in 25 mL of dry THF. The resulting mixture was refluxed for 24 h, ultimately affording a white product. The solid was filtered off and poured into an aqueous solution of saturated Na_2_CO_3_, and the resulting mixture was stirred. The product was filtered off, washed with dichloromethane, methanol, and water, and then dried at 100 °C for 3 h to afford a white solid (yield: 91%).

### Synthesis of TMU-27-NH_2_

A mixture of Zn(NO_3_)_2_·6H_2_O (0.027 g, 0.09 mmol), NH_2_-bdc (0.016 g, 0.09 mmol), bpipa (0.029 g, 0.09 mmol), and DMF (9 mL) was sonicated until all of the solids were uniformly dispersed and was then heated at 120 °C for 24 h. This afforded brown crystals as a pure phase, which were washed with DMF. Activation at 160 °C for 3 h led to solvent removal from the pores (yield: 64%). FT-IR (cm^–1^): 1670 (vs), 1564 (vs), 1509 (vs), 1433 (vs), 1330 (vs), 1303 (s), 1250 (s), 1207 (s), 1087 (m), 1064 (m), 1031 (m), 837 (vs), 770 (s), 597 (s), 526 (s). Elemental analysis calcd (%) for TMU-27-NH_2_: C 54.21, H 4.69, N 13.83; found: C 50.24, H 4.44, N 12.60.

### Synthesis of TMU-27

A mixture of Zn(NO_3_)_2_·6H_2_O (0.036 g, 0.2 mmol), bdc (0.020 g, 0.2 mmol), bpipa (0.038 g, 0.2 mmol), water (1.2 mL), acetic acid (160 μL), and DMF (10.8 mL) was sonicated until all of the solids were uniformly dispersed and was then heated at 85 °C for 24 h. This afforded white needle-shaped crystals, which were obtained as a pure phase and then washed with DMF. Activation at 160 °C under vacuum for 6 h led to solvent removal from the pores, resulting in the formation of the CP phase (yield: 71%). FT-IR (cm^–1^): 1673 (s), 1593 (vs), 1503 (vs), 1434 (m), 1385 (vs), 1332 (s), 1304 (s), 1233 (m), 1207 (s), 1032 (m), 837 (s), 748 (s), 597 (m), 537 (m). Elemental analysis calcd (%) for TMU-27: C 55.38, H 4.65, N 12.11; found: C 54.6, H 4.53, N 11.6

### Materials and Characterization

All commercially available reagents were used without further purification. Powder X-ray diffraction (PXRD) measurements were taken on an X’pert Pro MPD-Malvern PANalytical diffractometer with monochromatic Cu-Kα radiation (λCu = 1.5406 Å). Nonambient PXRD measurements were performed in a TTK600 Low-Temperature Chamber from Anton Paar, which was mounted inside the diffractometer, allowing for the use of a temperature range from −20 to 600 °C and in air/vacuum conditions. Fourier transform infrared (FT-IR) spectra were recorded on a Bruker Tensor 27FTIR spectrometer equipped with a Golden Gate, diamond, attenuated total reflection (ATR) cell in absorption mode at room temperature. Thermogravimetric analyses (TGA) were run in a PerkinElmer Pyris 1 under a N_2_ atmosphere and a heating rate of 10 °C/min. Scanning electron microscopy (SEM) was performed using a Quanta 650FEG SEM and a Magellan 400L XHR SEM. CO_2_- and N_2_-sorption isotherms were collected at 203 and 77 K, respectively, using an ASAP 2020 HD (Micromeritics). Temperature was controlled by using a liquid nitrogen bath (77 K) or a Lauda Proline RP 890 chiller (203–298 K). Total pore volumes (*V*_t_) were calculated at *P*/*P*_0_ = 0.95 (N_2_).

## Results and Discussion

**Figure 1:**
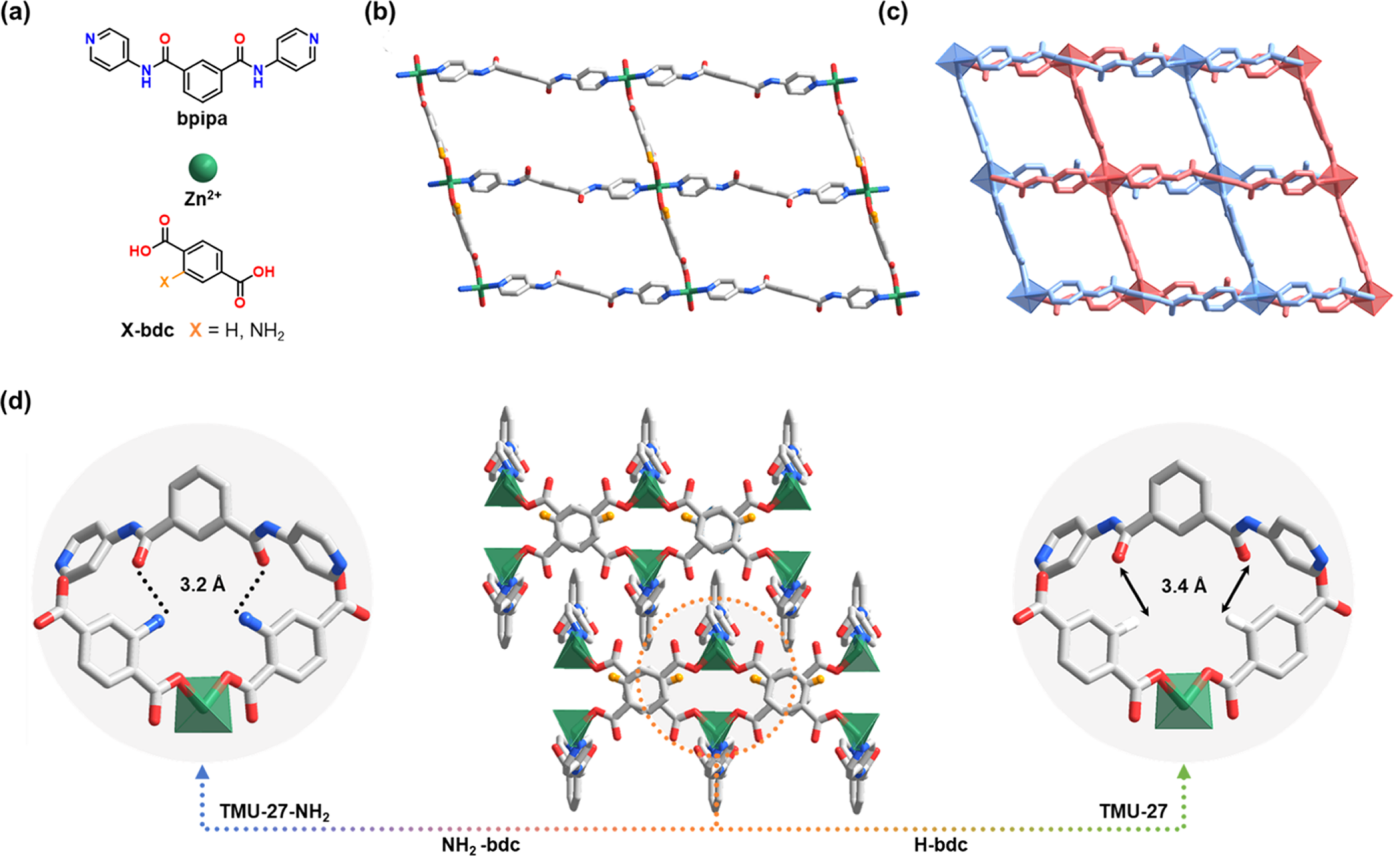
Synthesis and structural analysis of TMU-27 and TMU-27-NH_2_. (a) Representation of the respective molecular building units (TMU-27: X = H; TMU-27-NH_2_: X = NH_2_). (b) Representation of a single layer. (c) Interweaving layers, in which each layer is represented with a different color. (d) Interdigitation of the interweaving layers (center). Highlight of the hydrogen bonding between interdigitated layers in TMU-27-NH_2_ (left) in stark contrast to the lack of such bonding in TMU-27 (right).

**Figure 2:**
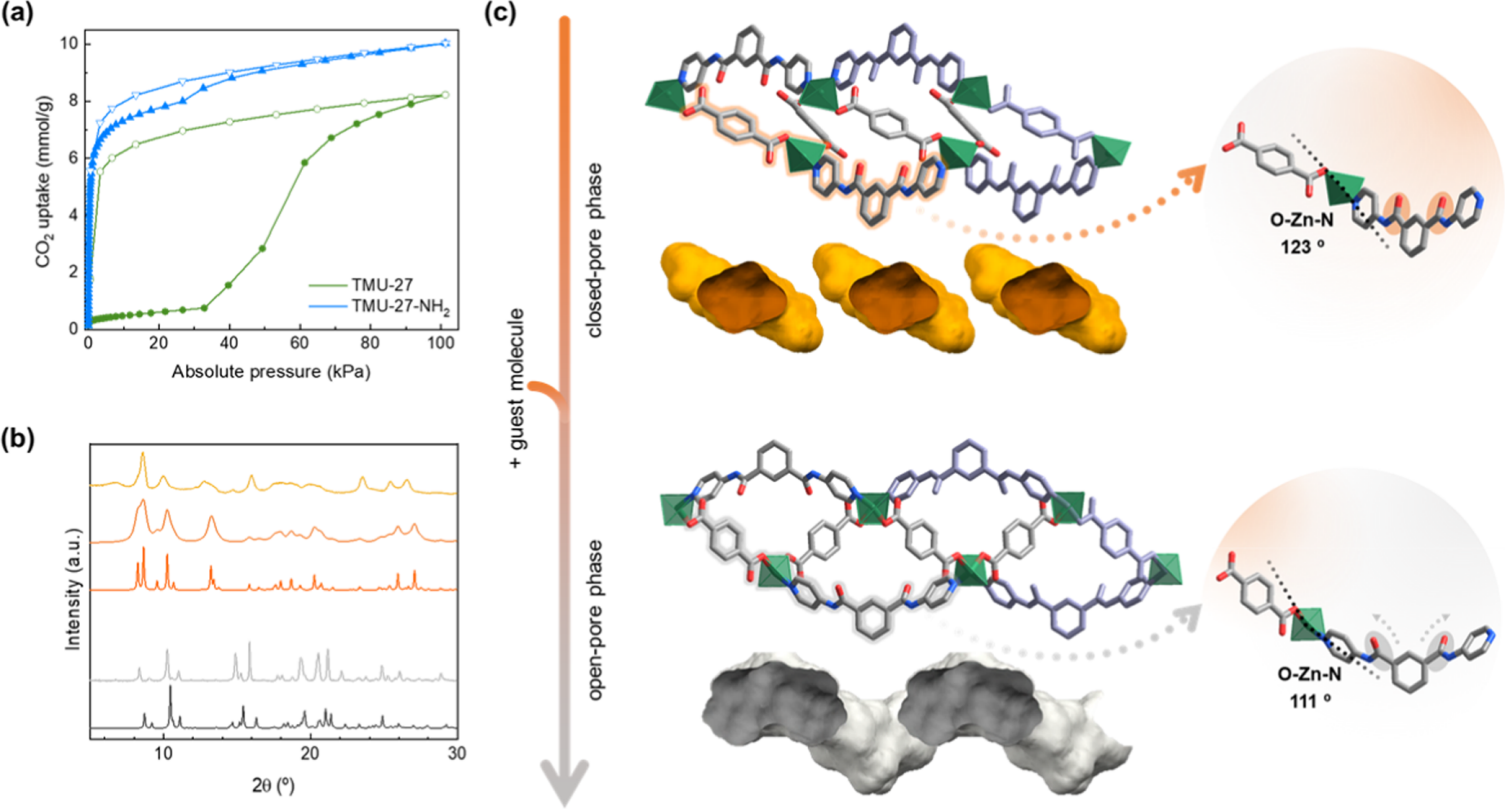
(a) Isotherms of the CO_2_ sorption at 203 K for TMU-27 (green) and TMU-27-NH_2_ (blue). Key: filled circles or triangles = adsorption branch; hollow circles or triangles = desorption branch. (b) Simulated (from SC-XRD) and experimental PXRD patterns for the closed-pore (CP) phase (top; dark orange = simulated; light orange = experimental, measured at 160 °C) and the open-pore (OP) phase (bottom; black = simulated; gray = experimental, measured at 160 °C) of TMU-27. To include disorder created in response to the removal of guest molecules in the CP phase, the FWHM of the simulated PXRD pattern (top, middle) was increased to 0.8·2θ, matching the experimental pattern. (c) Representation of the evolution from the CP phase to the OP phase in TMU-27.

We began with the synthesis of TMU-27. To this end, a mixture of Zn(NO_3_)_2_·6H_2_O, bpipa, and H_2_bdc in water, acetic acid, and DMF was heated at 85 °C for 1 day to afford white, needle-shaped crystals of TMU-27 (Figure 1a). The SC-XRD analysis of TMU-27 revealed that it crystallizes in a monoclinic lattice, with the *P*21/*c* space group (Table S1 in the SI). In this structure, each zinc ion is tetracoordinated in a slightly distorted tetrahedral geometry, bound to two N atoms on two bpipa linkers (Zn–N bond distance: 2.005(3) Å) and to two O atoms in monodentate carboxylate groups on two bdc linkers (Zn–O bond distance: 1.956(4) Å) (Figure S2a), leading to an overall 4-connected bidimensional network exhibiting an underlying **sql** topology (Figures 1b and S2c,d in the SI). Due to the alternating upward–downward orientation of the V-shaped bpipa ligand, the layers are interwoven (Figure 1c), leading to an overall stacking/interdigitating of interwoven 2D layers due to short contacts between the secondary amines from the bpipa ligand and the O atom from the bdc/NH_2_-bdc that does not participate in the metal–ligand coordinative bond. Zn equivalent positions are separated by a distance of 11.999(18) Å (Figure 1d), and this stacking results in the formation of one-dimensional (1D) open channels with a pore window size of 3.9 Å and a maximal pore diameter of 5.5 Å (Figures 2c and S3 in the SI).

Next, we synthesized TMU-27-NH_2_. Similarly to the previous reaction, a mixture of Zn(NO_3_)_2_·6H_2_O, bpipa, and NH_2_-bdc in DMF was heated at 120 °C for 1 day to afford rhombohedron-shaped brown crystals of TMU-27-NH_2_. Detailed crystallographic analysis revealed that it crystallizes in a monoclinic lattice with the *C*2/*c* space group and **sql** topology, similarly to TMU-27. Thus, TMU-27-NH_2_ is isostructural to TMU-27; however, it shows additional intermolecular hydrogen-bonding interactions [NH···O between the NH group from the NH_2_-bdc and the amide O atoms of bpipa; bond distance: 3.1 Å (Figure 1d)] between each pair of interwoven layers. Equivalent Zn positions in 2D-stacked layers are separated by a distance of 11.864(16) Å. The simulated PXRD patterns for TMU-27-NH_2_ and TMU-27 match with the corresponding patterns for the as-synthesized materials (Figures 2b and S4 in the SI). Also, their respective elemental analyses coincide with the expected formulas, indicating that the crystal structures are representative of the bulk materials.

Next, we evaluated the thermal stability of TMU-27 and TMU-27-NH_2_. Subsequent thermogravimetric analysis (TGA) revealed initial weight-loss values of 19.2% for TMU-27 and 19.8% for TMU-27-NH_2_, which we attributed to the loss of the guest solvent molecules (expected values: 21.0% for TMU-27, [Zn(bpipa)(bdc)]·2DMF, and 20.6% for TMU-27-NH_2_, [Zn(bpipa)(NH_2_-bdc)]·2DMF). Both frameworks began to decompose between 300 and 350 °C, suggesting high thermal stability for each (Figure S6 in the SI).

We then proceeded to perform CO_2_-sorption experiments on TMU-27 and TMU-27-NH_2_. Considering the thermal stabilities of these two MOFs, we activated them at 160 °C for either 6 h (TMU-27) or 3 h (TMU-27-NH_2_), prior to the CO_2_ sorption, which we measured at 203 K (Figure 2a). The desolvated phase of each MOF exhibited distinct CO_2_-driven adsorption properties. For TMU-27, the isotherm presents a sharp step at 32 kPa, corresponding to a type F-III adsorption isotherm (Figure 2a).^17^ Such distinctive isotherms are characteristic of flexible materials and exhibit a phase transformation from a closed-pore (CP) phase, which is formed upon desolvation/activation of the as-synthesized TMU-27 (Figure 2b), to an open-pore (OP) phase, upon adsorption of guest species (Figures 2c and S8 in the SI). Importantly, we confirmed the presence of such a CP phase in the desolvated TMU-27 by SC-XRD analysis. This phase has a triclinic lattice with the *P*1̅ space group. In comparison to the OP phase, the CP phase results from changes within the geometry of the bpipa ligand, which becomes planar, and a change in the bdc–Zn–bpipa angle, from 111° (OP) to 123° (CP), as shown in Figure 2c. These changes in the framework lead to disconnection of the 1D channels observed in the OP phase, resulting in the formation of a compartmentalized framework of discrete cavities (maximal pore diameter: 4.5 Å) and a simultaneous reduction of 23.2% in the unit-cell volume from the OP to the CP phases (Table S2 in the SI).

We observed that during the initial stage of adsorption, the CO_2_ molecules diffused into the discrete cavities of the CP phase, resulting in an uptake of 0.8 mmol/g, which corresponds to the formula [Zn(bpipa)(bdc)]·0.5CO_2_ (Figure 2a). Next, the internal pressure increased as the pores continued to fill, which eventually led to the migration of the CO_2_ molecules between cavities. Once the pressure surpassed 32 kPa, this induced structural changes within the framework, resulting in further adsorption of CO_2_. As previously demonstrated,^47^ this process may occur due to the elasticity of the framework, which in this case would be induced by the rotation of the bdc benzene ring, which would block access to the separate cavities. The maximal CO_2_ uptake that we observed for TMU-27 was 8.1 mmol/g at 101 kPa, which corresponds to the formula [Zn(bpipa)(bdc)]·3CO_2_. According to Gurvich’s rule, CO_2_ in the pores of the OP phase would have occupied a volume of 0.33 cm^3^/g. This value slightly exceeds the maximal theoretical pore volume of 0.31 cm^3^/g, as calculated based on the assumption that the probe radius corresponds to the size of one CO_2_ molecule.

Interestingly, the initial stage of the isotherm of TMU-27-NH_2_ follows a pattern typical of microporous rigid materials that exhibit a type I adsorption isotherm.^48^ In this case, CO_2_ molecules (kinetic diameter: 3.30 Å) efficiently diffused into the pores (window size: 4.2 Å), reaching a maximum uptake of 10.0 mmol/g at a pressure of 101 kPa. We observed that during desorption there was a slight hysteresis, which indicated a different desorption mechanism to that of adsorption. Structural analysis of the desolvated TMU-27-NH_2_ by SC-XRD proved the structure to be robust upon desolvation. Indeed, comparison of the pristine and desolvated TMU-27-NH_2_ revealed only a minor contraction in the distance between the NH_2_ group from the functionalized bdc linker and the O atom from the bpipa ligand: 3.2 Å in the pristine framework vs 3.1 Å in the desolvated form. These minor changes observed in the desolvated TMU-27-NH_2_ concluded in a minor reduction of 2% in the cell volume (Table S1 and Figure S7 in the SI). Thus, we reasoned that the hydrogen-bonding network resulting from the presence of NH_2_ from the functionalized bdc linker maintains the overall structure of the framework, effectively rigidifying it upon desolvation.

To further investigate the two isostructural MOFs, we measured their adsorption of N_2_ at 77K (Figure S9 in the SI). The CP phase of TMU-27 was almost nonporous for N_2_, showing that, in contrast to CO_2_, N_2_ cannot diffuse into the cavities and open the pores. Contrariwise, TMU-27-NH_2_ is porous to N_2_ and adsorbs 7.4 mmol/g at 95 kPa, presenting a Brunauer–Emmett–Teller (BET) area of 689 m^2^/g. These results further confirm the more rigid open structure of TMU-27-NH_2_ upon activation. Moreover, the experimental pore volume of 0.25 cm^3^/g for TMU-27-NH_2_ matches well to the calculated volume of 0.26 cm^3^/g. Again, we observed a different mechanism for desorption in TMU-27-NH_2_ than for adsorption: it appeared as a slight hysteresis, which, together with the aforementioned changes in the cell volume of TMU-27-NH_2_ during activation, indicated to us the existence of minor structural changes within the framework. However, we reasoned that these changes were substantially smaller than those in the case of TMU-27 due to the stabilizing effect of the hydrogen-bonding network (Table S1 and Figure S7 in the SI).

**Figure 3:**
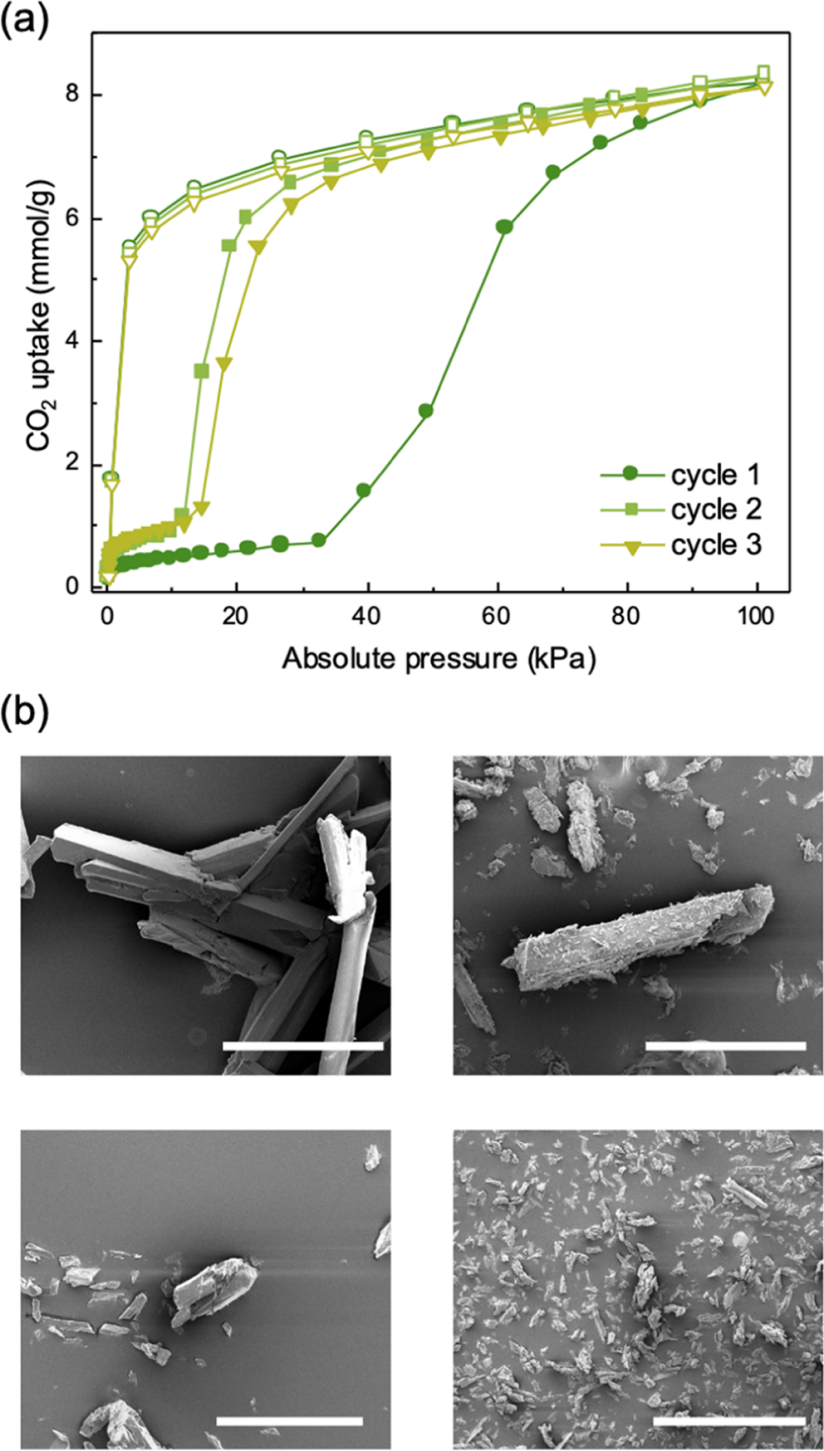
(a) Stability of the porosity of TMU-27 upon repeated cycles of CO_2_ adsorption (solid symbols) and desorption (hollow symbols) at 203 K. (b) Scanning electron micrographs of the as-synthesized (top, left) and desolvated (top, right) TMU-27 as compared to TMU-27 after the first (bottom, left) and second (bottom, right) CO_2_ adsorption/desorption cycle. Scale bars: 100 μm.

A critical and highly desirable feature of flexible MOFs is the reversibility of adsorption properties in cyclic experiments.^43^ Thus, to further investigate the behavior of TMU-27, we measured its response to repeated cycles of CO_2_ adsorption/desorption, which we monitored by SEM (Figure 3). Although the successive cycles exhibited comparable step-containment behavior, there was a remarkable shift in the gate-opening pressure from 32 kPa in the first isotherm down to 11 kPa in the second isotherm and, finally, up to 13 kPa in the third. Irrespective of the number of cycles, the total uptake of CO_2_ by TMU-27 at 203 K remained constant: 8.1 mmol/g at 101 kPa, demonstrating that this MOF does indeed exhibit the reversibility of adsorption–desorption cycles under repeated stress. As revealed by SEM imaging, the adsorption/desorption stress led to milling of the samples (Figure 3b), which underwent a considerable reduction in crystal size from >100 μm down to 55–65 μm (close to a 2-fold reduction) in the second isotherm and further down to 25–35 μm (close to a 5-fold reduction) in the third. This effect might lead to greater accessibility of CO_2_ to the framework and to lower diffusion resistance due to a higher surface-area-to-volume ratio.^49^ Nevertheless, considering that type F-III adsorption isotherms remained in subsequent sorption cycles, we deduced that this effect is insufficient to abrogate the flexibility of TMU-27.

## Conclusions

Here, we have reported the synthesis of two 2D interweaving Zn-**sql**-MOFs, TMU-27 and TMU-27-NH_2_, which differ in the substitution of their respective bdc linkers: an H atom in TMU-27 vs an NH_2_ group in TMU-27-NH_2_. While TMU-27 is nonporous to N_2_ but highly flexible upon CO_2_ sorption/desorption, TMU-27-NH_2_ exhibits adsorption properties commonly found in rigid MOFs. In TMU-27-NH_2_, the introduction of an amino group stabilizes the two-dimensional interweaving layers through hydrogen bonding. This hydrogen bonding prevents TMU-27-NH_2_ to be flexible as it retains its original open-pore structure upon activation, making it porous to, for example, N_2_ and CO_2_. Flexibility in TMU-27 undergoes a change from an open-pore (OP) phase to a closed-pore (CP) phase upon activation. This flexibility is reactivated upon CO_2_ adsorption, in which CO_2_ can diffuse into the discrete cavities of the CP phase, resulting in the formation of a pore opening in the OP phase. TMU-27 returns to its CP phase upon CO_2_ desorption. We elucidated these structural transformations of TMU-27 by SC- and PXRD studies and then through repeated CO_2_-sorption measurements. When exposed to adsorption/desorption stress, TMU-27 undergoes a significant reduction in crystal size, resulting in a shift in the gate-opening pressure from 32 to ∼12 kPa; however, the total CO_2_ uptake (8.1 mmol/g) remains constant. Therefore, the transition mechanism of adsorption does not change, but the energy of transformation decreases with a reduction in the crystal size. In conclusion, we have presented how the flexibility of a MOF can be modulated through induction of linker-dependent hydrogen bonding. Moreover, we are confident that our findings should inform future work on how the crystal size of stimuli-responsive porous materials influences their adsorption properties.

## Supporting Information Available

Additional experimental details and materials and methods, including ^1^H NMR of the ligand synthesis, X-ray crystallography and crystal data, FTIR, TGA, reversibility test, and N_2_ adsorption (PDF)
