# Supplementary material for: Modulation of the Dynamics of a Two-Dimensional Interweaving Metal–Organic Framework through Induced Hydrogen Bonding
Source: Inorg Chem. 2024 Mar 14;63(12):5552–8. doi: 10.1021/acs.inorgchem.3c04522 (PMC10966731; doi:10.1021/acs.inorgchem.3c04522)
Supplement: Supplementary file 1 — ic3c04522_si_001.pdf [file ic3c04522_si_001.pdf]

Supplementary Information

Modulation of the dynamics of a two-dimensional interweaving metal-organic framework through induced hydrogen-bonding

Pilar Fernández-Seriñán,[a,b] Kornel Roztocki,[c] Vahid Safarifard,[d] Vincent Guillerm,[a] Sabina Rodríguez-Hermida,[a] Judith Juanhuix,[e] Inhar Imaz,[a,b,*] Ali Morsali,[f,*] and Daniel Maspoch[a,b,g,*]

[a.] Catalan Institute of Nanoscience and Nanotechnology (ICN2), CSIC and The Barcelona Institute of Science and Technology, Campus UAB, Bellaterra, 08193 Barcelona, Spain

[b.] Chemistry Department of Autonomous University of Barcelona (UAB), Campus UAB, Bellaterra, 08193 Barcelona, Spain

[c.] Faculty of Chemistry, Adam Mickiewicz University, Uniwersytetu Poznańskiego 8, 61-614 Poznań, Poland

[d.] Department of Chemistry, Iran University of Science and Technology, Tehran 16846-13114, Iran

[e.] ALBA Synchrotron, Cerdanyola del Vallès, Barcelona 08290, Spain

[f.] Department of Chemistry, Faculty of Sciences, Tarbiat Modares University, P.O. Box 14115-175, Tehran, Iran

[g.] ICREA, Pg. Lluís Companys 23, 08010 Barcelona, Spain

e-mail of corresponding authors:

daniel.maspoch@icn2.cat

inhar.imaz@icn2.cat

morsali_a@modares.ac.ir

Table of Contents

S1. ^{1}H-NMR 2

S2. Single-crystal X-ray diffraction 3

S3. Powder X-ray diffraction 6

S4. Infrared spectroscopy (IR) 7

S5. Thermogravimetric analysis (TGA) 8

S6. Crystal structure of TMU-27-NH_2 9

S7. Unit Cells comparison 10

S8. Reversibility test 11

S9. N_2 adsorption 12

S9. References 13

S1. ^{1}H-NMR

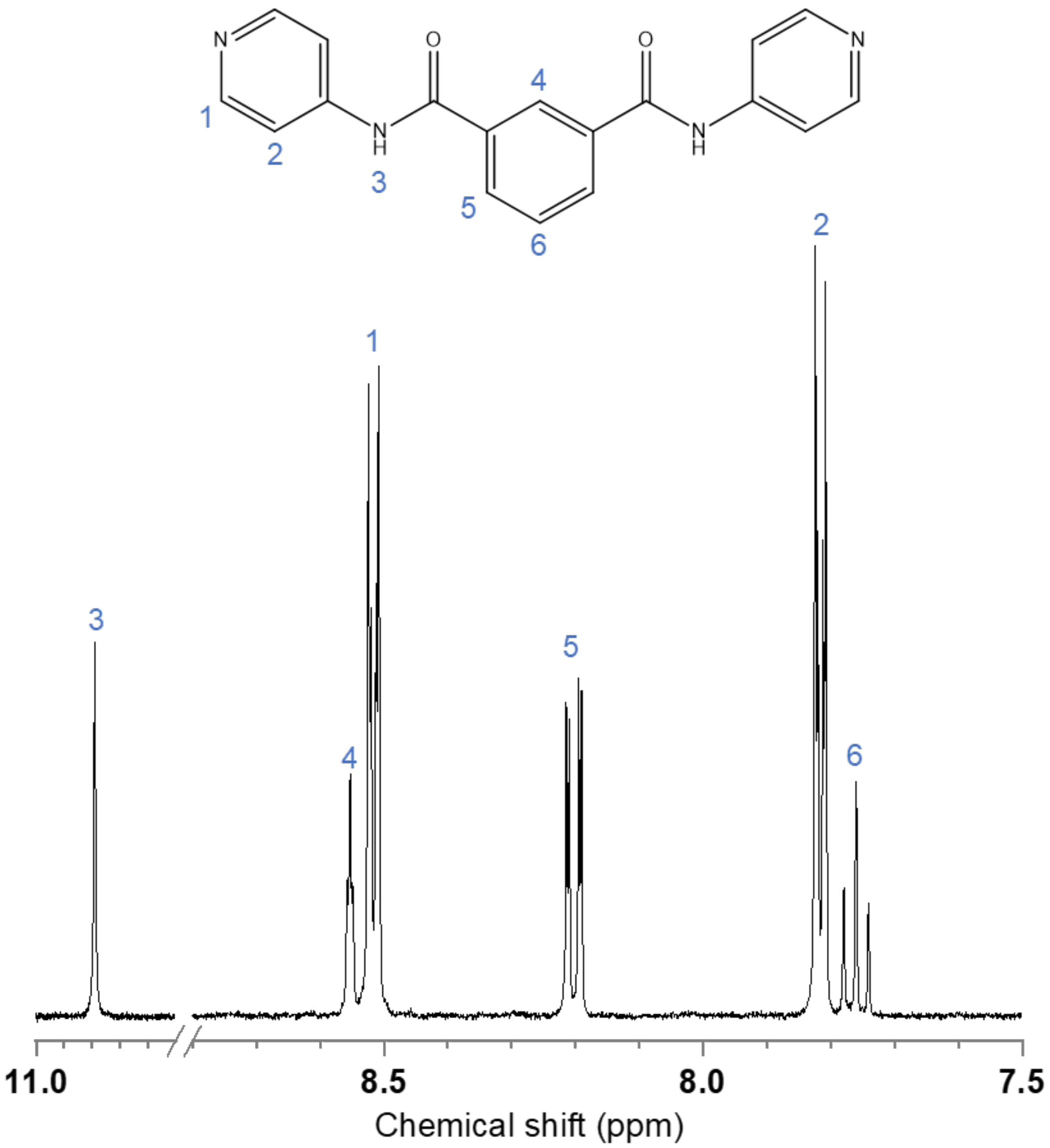

Figure S1. H^{1}NMR spectrum (400 MHz) of the synthesised ligand N,N'-bis-4-pyridyl-isophthalamide (bpipa).

S2. Single-crystal X-ray diffraction

Crystallographic data for TMU-27-op, TMU-27-cp and TMU-27-NH_2-act were collected at 100K on the XALOC beamline at ALBA synchrotron (λ = 0.82653 Å).[1] Data from TMU-27-op were indexed, integrated, and scaled using the XDS[2] and iMOSFLM[3] programs, whereas TMU-27-cp and TMU-27-NH_2-act data were indexed, integrated, and scaled using the Xia2 package.[4] Absorption correction was not applied. Single-Crystal X-Ray Diffraction (SC-XRD) for TMU-27-NH_2 was collected at 293 K on a Bruker AXS SMART Apex diffractometer, using graphite monochromated Mo-Kα radiation (λ = 0.71073 Å), and were corrected for Lorentz and polarisation effects. The frames were integrated with the Bruker SAINT[5] software package. Absorption corrections were applied using the program SADABS[6], giving max./min. transmission factors of 1.000/0.4760. The structures were solved by direct methods and subsequently refined by correction of F^2 against all reflections, using SHELXS2013[7] and SHELXL2013[8] within the WinGX package (for TMU-27-NH_2 and TMU-27 op)[9] and Olex2 package (for TMU-27-cp and TMU-27-NH $_2$-act)[10] All non-hydrogen atoms were refined with anisotropic thermal parameters by full-matrix least-squares calculations on F^2, using the program SHELXL2013. Hydrogen atoms were inserted at calculated positions and constrained with isotropic thermal parameters, except for the hydrogen atoms of the NH and NH_2 groups in TMU-27-NH_2 and TMU-27-NH_2-act, which were located from the difference Fourier map. In TMU-27-NH_2, the oxygen atom of the carbonyl group is distorted in two positions, at 70% and 30% occupancy. The contributions of two DMF molecules in TMU-27-NH_2 (302 electrons per unit cell) to the diffraction pattern could not be rigorously included in the model and thus, were consequently removed with the SQUEEZE routine of PLATON.[11]

Table S1. Crystal data and structure refinement data for TMU-27 and TMU-27-NH_2, both in as-synthesised and activated forms. Act: activated; CP: closed-pore phase; OP: open-pore phase.

Compound	**TMU-27 OP**	**TMU-27 CP**	**TMU-27-NH_2**	**TMU-27-NH_2 Act**
Empirical formula	$C_{64}H_{64}N_{12}O_{16}Zn_2$	$C_{48}H_{38}N_8O_{12}Zn_2$	$C_{32}H_{33}N_7O_8Zn$	$C_{26}H_{20}N_5O_6Zn$
Formula weight	1388.01	1049.64	709.02	563.84
Crystal system	Monoclinic	Triclinic	Monoclinic	Monoclinic
Space group	$P2_1/c$	P -1	$C2/c$	$C2/c$
CCDC ref	2302108	2302107	2302105	2302106
a (Å)	10.910(5)	10.6757(16)	11.1652(17)	11.1892(2)
b (Å)	40.570(4)	11.4086(14)	20.937(3)	20.2936(4)
c (Å)	15.277(4)	11.6434(13)	15.066(2)	15.1957(3)
α (deg)	90	106.795(9)	90	90
β (deg)	93.100(10)	102.191(11)	93.054(3)	94.025(2)
γ (deg)	90	97.436(11)	90	90
V (Å^3)	6751.99	1299.24(24)	3517.0(9)	3441.96
Z	4	2	4	4
F (000)	2880	591	1472	1156
θ range (°)	1.167-33.831	4.4-41.64	1.945-28.324	2.422-33.702
Ind refln (R_{int})	13454(0.0771)	2570(0.10322)	4051 (0.0382)	3656(0.0890)
Final R indices [I > 2σ(I)]	R1 = 0.0795 wR2 = 0.22233	R1 = 0.1893 wR2 = 0.4955	R1 = 0.0851 wR2 = 0.2417	R1 = 0.0691 wR2 = 0.2036

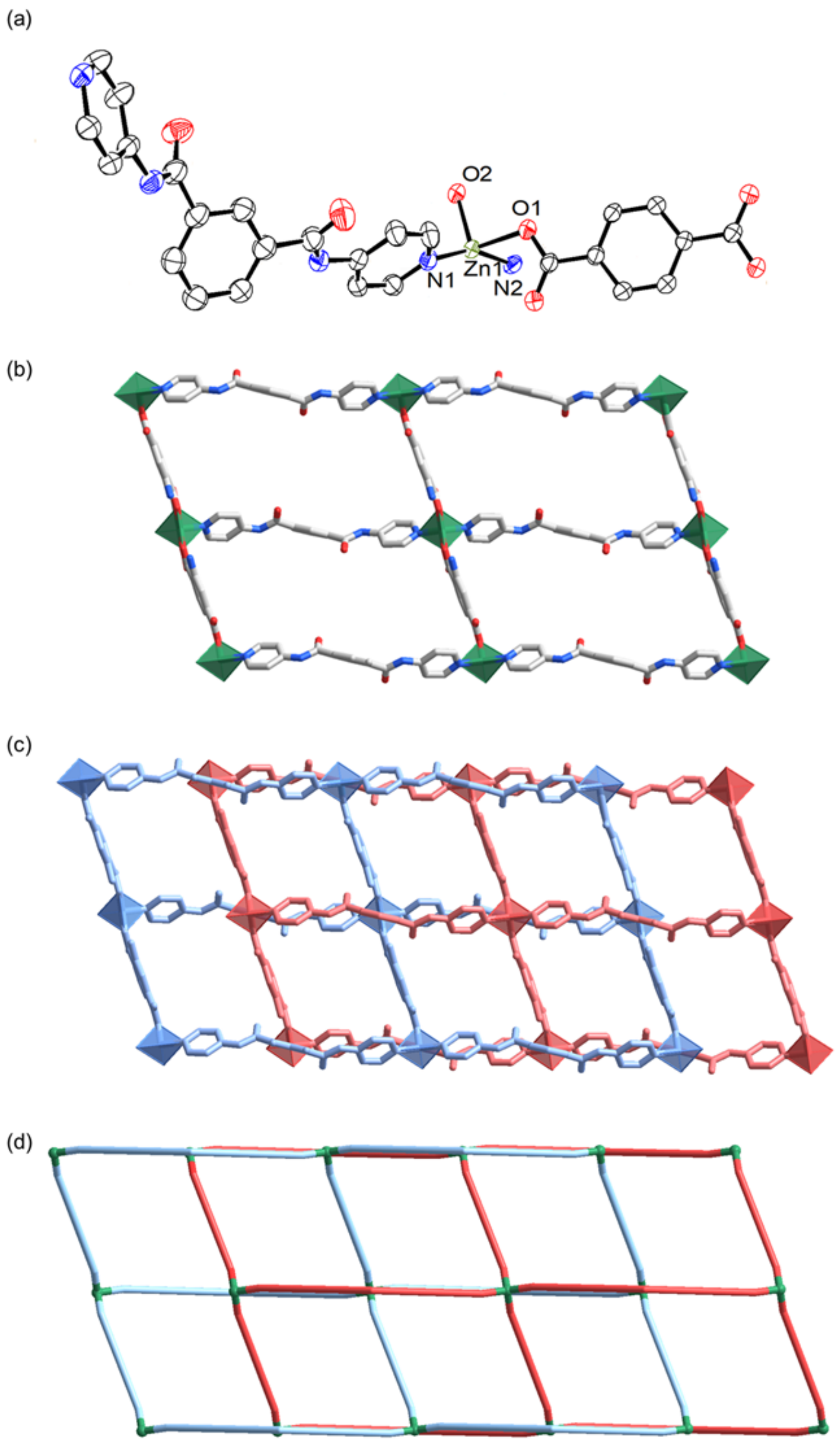

Figure S2. (a) Ortep diagram depicting the coordination motif between the metal node and the ligands. The ellipsoids are drawn at the 50% probability level. Hydrogen atoms are omitted and only the coordination environment of Zn(II) has been labelled for the sake of clarity. (b) Single layer. (c) Interwoven layers of TMU-27 and TMU-27-NH_2 and (d) underlying sql topology, where Zn(II) is highlighted in green.

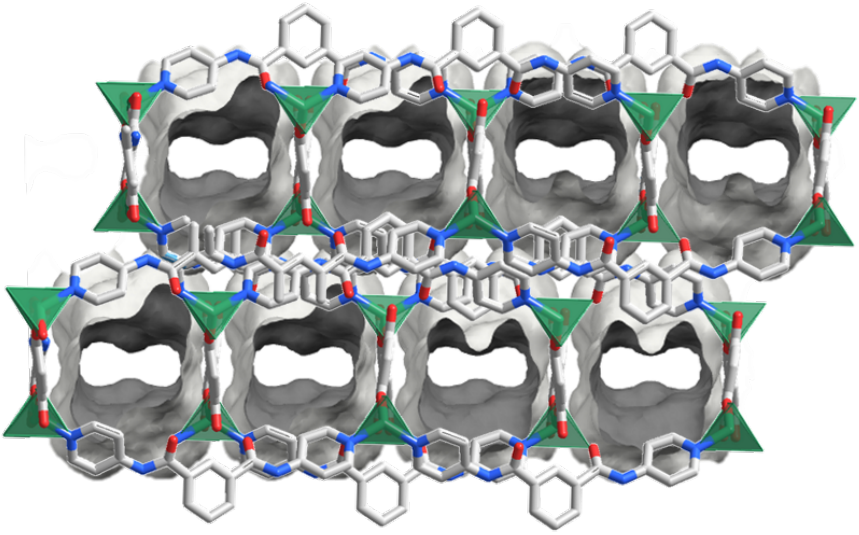

Figure S3. One-dimensional channels in the Open-Pore (OP) phase of TMU-27.

S3. Powder X-ray diffraction

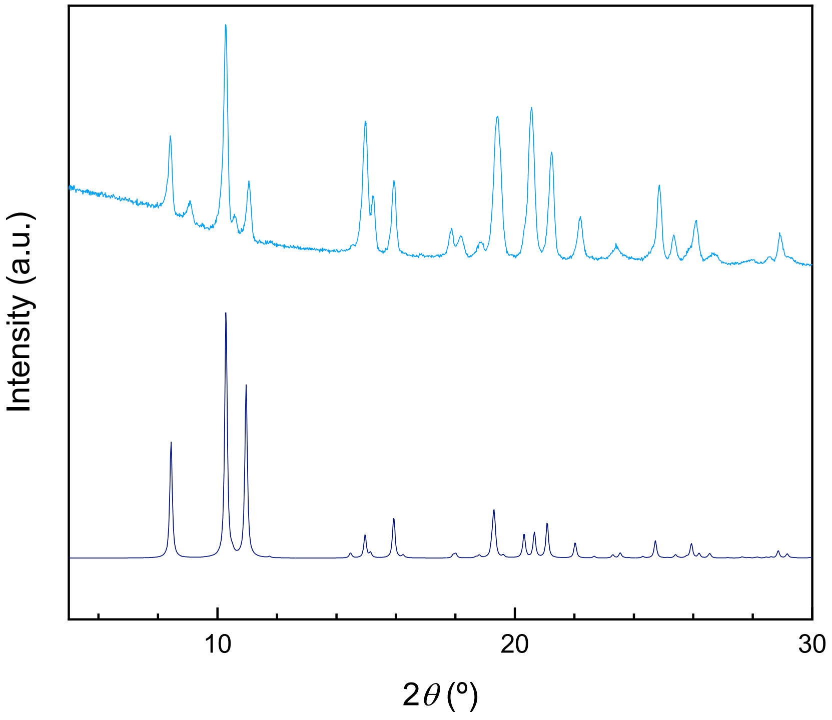

Figure S4. Experimental PXRD pattern for as-synthesised TMU-27-NH_2 (top, blue) and its corresponding simulated PXRD pattern (purple, bottom). The additional peak at 8.943º (identified with the asterisk) corresponds to DMF molecules trapped inside the framework that is not present in the simulated pattern as the structure was squeezed during the refinement.

S4. Infrared spectroscopy (IR)

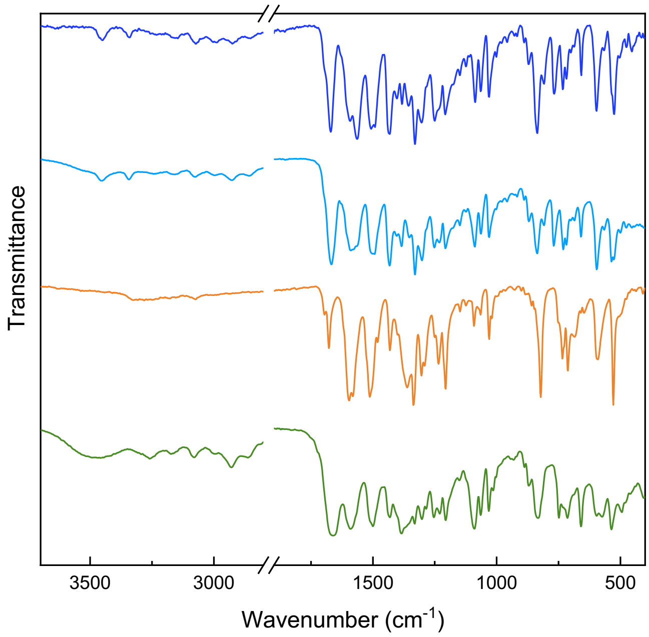

Figure S5. IR spectra of the as-synthesised Open-Pore (OP) phase of TMU-27 (green), activated Closed-Pore (CP) phase of activated TMU-27 (orange), as-synthesised TMU-27-NH_2 (light blue), and activated TMU-27-NH_2 (dark blue).

S5. Thermogravimetric analysis (TGA)

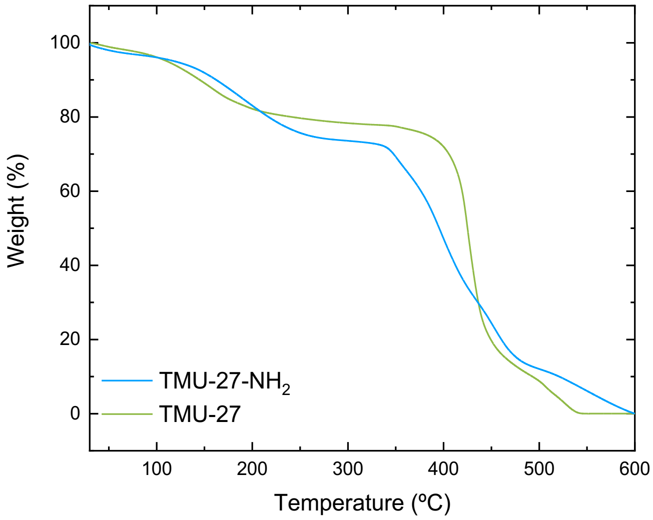

Figure S6. Thermogravimetric traces for as-synthesised TMU-27 (green) and TMU-27-NH_2 (blue).

S6. Crystal structure of TMU-27-NH_2

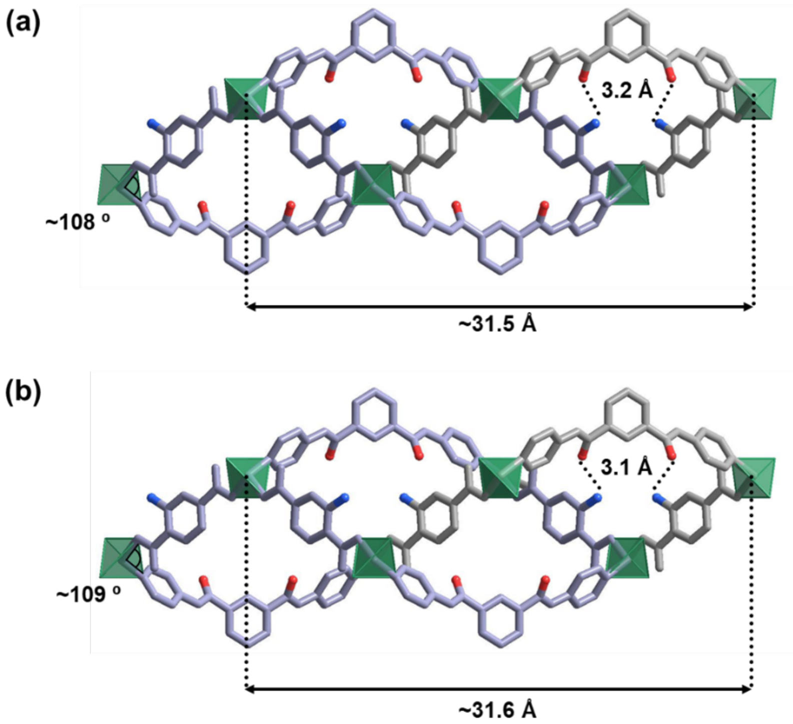

Figure S7. Crystal structures of as-synthesized TMU-27-NH_2 (a) and TMU-27-NH_2 that had been activated at 160 ºC for 3 h (b).

S7. Unit Cells comparison

For comparison, the unit cell of Open-Pore (OP) phase TMU-27 was transformed into a triclinic lattice. For valid crystallographic information, the Single Crystal X-Ray Diffraction data for these materials can be found in Table S1.

Table S2. Comparison of the unit cell parameters of TMU-27 in Open-Pore (OP) phase and Closed-Pore (CP) phase.

	a (Å)	*b* (Å)	*c* (Å)	*α* (°)	*β* (°)	*γ* (°)	*V* ($Å^3$)
TMU-27 OP	11.143	15.277	10.910	93.100	65.915	93.573	1691.4
TMU-27 CP	10.676	11.409	11.644	106.795	102.191	97.436	1299.2

S8. Reversibility test

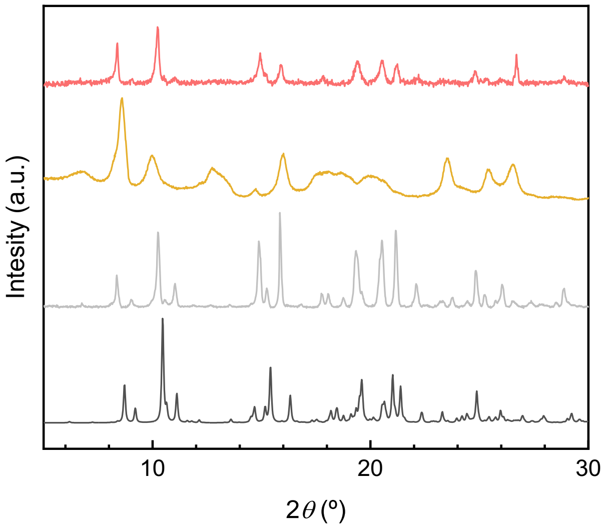

Figure S8. Powder X-Ray Diffraction patterns for the simulated Open-Pore (OP) phase of TMU-27 (bottom, black), as-synthesised TMU-27 (second to bottom, light grey), activated Closed-Pore (CP) phase of TMU-27 (second to top, orange), and after immersion of the activated crystal in DMF for 2 h (top, red).

S9. N_2 adsorption

(a)

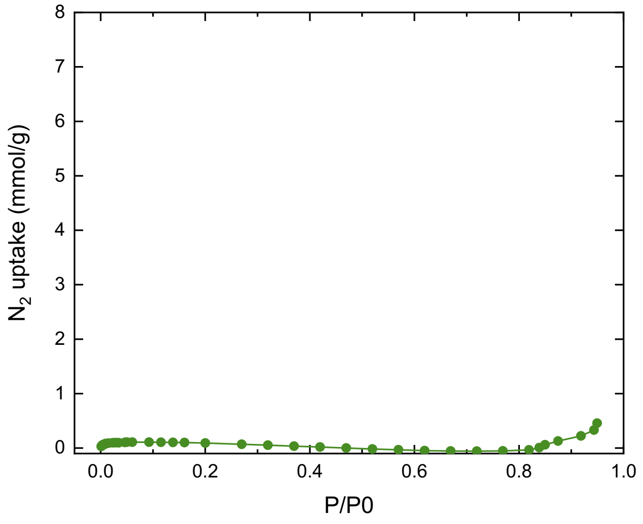

(b)

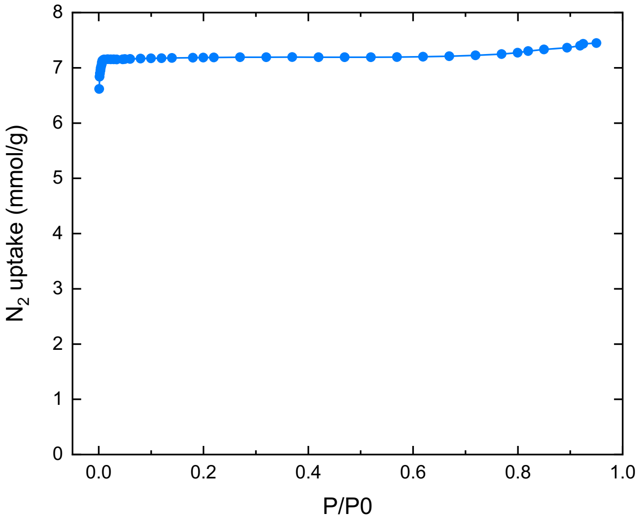

Figure S9. N_2-sorption isotherms at 77 K for TMU-27 (a) and TMU-27-NH_2 (b). Filled circles: adsorption branch; Hollow circles: desorption branch.

S9. References

1. J. Juanhuix, F. Gil-Ortiz, G. Cuni, C. Colldelram, J. Nicolas, J. Lidon, E. Boter, C. Ruget, S. Ferrer and J. Benach, Developments in optics and performance at BL13-XALOC, the macromolecular crystallography beamline at the Alba Synchrotron, *J. Synchrotron Radiat.*, 2014, **21**, 679-689.
2. W. Kabsch, Integration, scaling, space-group assignment and post-refinement, *Acta Cryst. D*, 2010, **66**, 133-144.
3. A. Leslie, The integration of macromolecular diffraction data, *Acta Cryst. D*, 2006, **62**, 48-57.
4. G. Winter, xia2: an expert system for macromolecular crystallography data reduction, *J. Appl. Cryst.*,2010, **43**, 186-190.
5. Siemens SAINT, 4; Siemens Analytical X-ray Instruments Inc.: Madrison, Wisconsin, USA, 1996.
6. L. Krause, R. Herbst-Irmer, G. M. Sheldrick and D. Stalke, Comparison of silver and molybdenum microfocus X-ray sources for single-crystal structure determination, *J. Appl. Crystallogr.*, 2015, **48**, 3-10.
7. G. M. Sheldrick, Z. Dauter, K. S. Wilson, H. Hope and L. C. Sieker, The application of direct methods and Patterson interpretation to high-resolution native protein data, *Acta Cryst. D*, 1993, **49**, 18-23.
8. G. Sheldrick, Crystal structure refinement with SHELXL, *Acta Cryst. C*, 2015, **71**, 3-8.
9. L. Farrugia, WinGX and ORTEP for Windows: an update, *J. Appl. Crystallogr.*, 2012, **45**, 849-854.
10. O. V. Dolomanov, L.J. Bourhis, R.J. Gildea, J. A. K. Howard, H. Puschmann, OLEX2: a complete structure solution, refinement and analysis program, J. Appl. Crystallogr., 2009, **42**, 339-341.
11. A. L. Spek, PLATON. A Multipurpose Crystallographic Tool, http://www.platonsoft.nl/platon/pl000000.html).
